# Diazepam alters the shape of alpha oscillations recorded from human cortex using EEG

**DOI:** 10.1162/IMAG.a.1169

**Published:** 2026-03-12

**Authors:** George M. Opie, Natalie Schaworonkow, Pedro C. Gordon, Dania Humaidan, Ulf Ziemann

**Affiliations:** Discipline of Physiology, School of Biomedicine, The University of Adelaide, Adelaide, Australia; Ernst Strüngmann Institute for Neuroscience in Cooperation with Max Planck Society, Frankfurt am Main, Germany; Department of Neurology & Stroke, Eberhard Karls University of Tübingen, Tübingen, Germany; Hertie-Institute for Clinical Brain Research, Eberhard Karls University of Tübingen, Tübingen, Germany

**Keywords:** electroencephalography, alpha oscillation, waveform shape, diazepam, GABAA receptor, pharmaco-EEG

## Abstract

While neural oscillations are conventionally assessed via their frequency, power and phase, developing literature suggests that their shape also provides neurophysiological and functional information. However, the extent to which the shape of oscillations recorded non-invasively in humans index specific brain processes remains unclear. This study implemented a pharmaco-EEG approach to begin addressing this limitation. Resting-state EEG data were collected before and after placebo or diazepam, a positive allosteric modulator of type A γ-aminobutyric acid (GABA_A_) receptors, with 15 participants included in the main analysis. The shape of individual cycles in the alpha band was then derived using empirical mode decomposition, followed by extraction of principal components (PCs) describing specific facets of alpha shape. Results of this approach show that all shape features were unchanged following placebo. In contrast, diazepam was associated with complex changes in several shape features, including peak-trough shape and edge speed. While changes in shape were apparent in all cortical lobes, the strongest alterations were specific to sensorimotor and parietal cortices. Taken together, our results support the neurophysiological utility of waveform shape, particularly with respect to non-invasive human recordings. Furthermore, the regional specificity of effects highlights the need for more granular exploration of waveform diversity.

## 1 Introduction

Neural oscillations represent a stereotypical feature of the brain’s electrical activity (Buzsáki & Draguhn, 2004). These rhythmic fluctuations in voltage occur across a broad frequency range and are ubiquitous throughout the brain. Furthermore, they have been associated with critical brain functions such as inter-areal communication and signal integration (Fries, 2005; Jensen & Colgin, 2007), and therefore underpin everyday processes like learning and memory (Buzsaki, 2006; Buzsáki & Watson, 2012). Alterations in oscillatory activity have consequently been associated with numerous pathologies and disorders, including schizophrenia (Uhlhaas & Singer, 2010), ADHD (Calderone et al., 2014), and Parkinson’s disease (Oswal et al., 2013). Given the above, it is unsurprising that the examination of neural oscillations represents the focus of a huge literature. From a methodological perspective, this has involved an expansive suite of approaches. However, derivation of oscillatory power via Fourier-based techniques has been the mainstay; this approach has provided a powerful means to investigate complex oscillatory activity in many different contexts, and has driven countless neuroscientific discoveries.

While spectral decomposition via Fourier-based methods captures some important features of neural oscillatory activity, it disregards others. In particular, the underlying assumption that a sinusoidal waveform best replicates endogenous oscillatory activity ignores the well-established fact that many oscillations are distinctly non-sinusoidal (for review, see: Cole & Voytek, 2017). Acceptance of this approximation has been driven by the belief that idiosyncrasies in oscillation shape do not provide any information about underlying generative processes. However, a growing body of evidence provides evidence to the contrary. For example: the shape of delta oscillations recorded in rat motor cortex are altered following lesion of dopaminergic cells in basal ganglia (Parr-Brownlie et al., 2022); non-sinusoidal features of theta cycles recorded from rat hippocampus reflect synchronization, activation sequence, and firing rate of pyramidal neurons and interneurons (Cole & Voytek, 2018) and have been associated with running speed (Ghosh et al., 2020; Quinn, Lopes-dos-Santos, Huang, et al., 2021); the shape of sensorimotor beta oscillations reflects thalamic input to cortical areas (Bonaiuto et al., 2021; Sherman et al., 2016), and shows increased sharpness in Parkinson’s patients, with changes correlating to limb rigidity (O’Keeffe et al., 2020) and being normalized by treatment (Cole et al., 2017; Jackson et al., 2019). Consequently, non-sinusoidal features of neural oscillations can index physiological information that is functionally and clinically relevant.

Although the developing literature demonstrates the utility of oscillation shape as an index of neural function, our understanding of the associated physiological processes is largely driven by invasive techniques, animal models, or computational methods. In contrast, interpretation of shape within other contexts, such as human electro- (EEG) or magnetoencephalography (MEG) recordings, remains unclear. Given that these represent more commonly applied electrophysiological measures of human brain activity, this represents a significant limitation to the broader application of shape metrics. One technique that has been effective for addressing this issue is to use pharmacological intervention to modulate neurotransmission through specific neuronal receptors, and record associated changes in EEG/MEG recordings. Within this pharmaco-M/EEG approach, drug-related changes in the outcome measure suggest generative contributions from the targeted receptor. This approach has underpinned identification of the role played by key neurotransmitters (e.g., γ-aminobutyric acid [GABA] and glutamate) and ions (e.g., calcium, sodium and potassium) in several electrophysiological measures (e.g., Darmani & Ziemann, 2019; Mucci et al., 2006; Muthukumaraswamy, 2014; Purdon et al., 2015).

The main aim of the current study was, therefore, to use pharmacological intervention targeting type A GABA receptors (GABA_A_) to investigate the neurophysiological processes that contribute to the non-sinusoidal shape of neural oscillations. To mitigate methodological challenges associated with detecting waveform shape differences in EEG data, we focused on oscillations in the alpha band (7–14 Hz). This was motivated by several factors: first, alpha is predominant across individuals; second, alpha presents with large signal-to-noise ratio (SNR), and well established non-sinusoidal shape; third, the effects of GABAergic modulation on alpha power have been well established (for review, see: Lozano-Soldevilla, 2018); fourth, alpha activity is more reliably discernible than other commonly quantified rhythms, such as beta (challenging to quantify in the presence of alpha harmonics: Schaworonkow, 2023) and theta (not sufficiently present in resting recordings, in our experience). Subsequently, alpha shape was examined in healthy participants before and after oral intake of diazepam, a positive allosteric modulator of GABA_A_ receptors, using a randomized placebo-controlled double-blind crossover design. Given recent evidence showing that several spatially distinct alpha oscillations can be identified based on their shape (Bender et al., 2025; Giehl & Siegel, 2024), effects of diazepam were contrasted between alpha rhythms originating from different cortical areas. The alpha rhythms across different areas are sometimes referred to by more specific terms, (e.g., mu for rhythms originating from sensorimotor regions, tau for rhythms originating in the auditory-related regions). For conciseness, we refer to all rhythm types collectively using the term ‘alpha rhythm’.

## 2 Methods

### 2.1 Dataset and participants

The current study utilized resting-state EEG data that were collected but not examined during a previously published project (Gordon et al., 2023). While experimental information relevant to the current study is reported here, the reader is referred to the previous publication for additional details about the original protocol. Recordings were collected from 21 healthy, right-handed participants aged between 18 and 50 years (mean age ± SD = 25.5 ± 4.7, 14 female). Exclusion criteria included use of centrally acting medication, history of psychiatric or neurological disease, history of alcohol or illicit drug use, and current pregnancy or breastfeeding. Written, informed consent was required prior to inclusion; all experimentation was conducted in accordance with the Declaration of Helsinki; and the study was approved by the ethics committee of the medical faculty of the University of Tübingen (approval number 456/2019BO2). As data from 6 participants failed to meet source matching and alpha SNR exclusion criteria (detailed below), a total of 15 participants were included in the analysis: 12 of these contributed data to both sessions, 3 contributed data to the placebo session only, and 3 contributed data to the diazepam session only (see Supplementary Table S1).

### 2.2 Experimental arrangement

Participants attended two experimental sessions held at least 1 week apart, during which they sat comfortably on a reclining chair in a quiet room. Each session involved recording of eyes-open, resting-state EEG before and after oral intake of either 20 mg diazepam (diazepam; Ratiopharm) or placebo (placebo; P-Tabletten Lichtenstein). The order of diazepam and placebo sessions was pseudorandomized between participants, and both participants and experimenters were blinded to the nature of the intervention for each session. This was facilitated by the drugs having comparable appearance and being labeled with identifying codes that were only revealed following study completion. Drug intake occurred immediately following baseline EEG recording, with post-intervention EEG being collected 60 mins after drug ingestion to allow diazepam to reach peak plasma concentrations (Shader et al., 1984). Scalp EEG was recorded using an EasyCap EEG cap (Brain Products, GmbH, Germany), with 64 Ag-AgCl sintered ring electrodes (standard 10-10 locations) connected to a NeurOne amplifier (Bittium, Finland). Recordings were referenced to FCz, the ground electrode was positioned at PPO1h (centered between P1-Pz and PO3-POz), and impedance was maintained < 5 kΩ. All recordings were filtered on-line (0.16 Hz–1.25 kHz) and digitized at 5 kHz prior to storage for off-line analysis. Five minutes of resting-state data were collected at each time point, during which participants fixated on a black cross displayed 1 m in front of them.

### 2.3 Data analysis

#### 2.3.1 EEG preprocessing

**Fig. 1.**
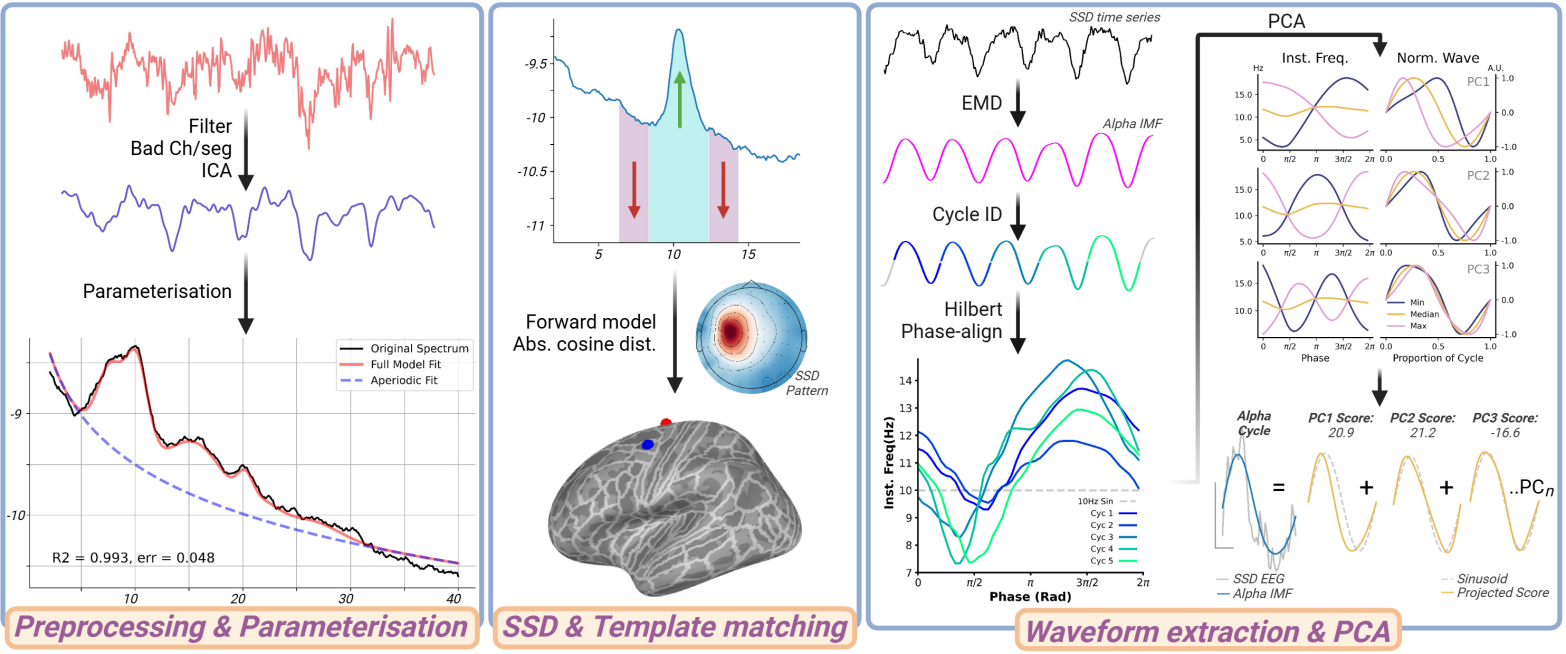
Analysis pipeline. Left*:* Minimal preprocessing was applied prior to spectral parameterization to quantify individual alpha peak frequency. Middle: Application of spatio-spectral decomposition (SSD) increased alpha band SNR, prior to identification of component sources with template matching. Right: Empirical mode decomposition (EMD) application extracted narrowband alpha modes, followed by cycle identification, derivation of phase-aligned instantaneous frequency, and application of principal component analysis (PCA). Derived scores for each cycle describe the extent to which different shape dimensions are expressed.

Data were down-sampled to 1000 Hz and line noise removed using the zap-line plugin for EEGLAB (v2024.0) (Delorme & Makeig, 2004) on the Matlab platform (R2021b, MathWorks, USA). Data were then exported to MNE-Python (v1.8.0) (Gramfort et al., 2013; Larson et al., 2024), where band-pass filtering (1–100 Hz) was applied using the *filter* method for raw objects, followed by identification of bad channels using the *find_all_bads* method of the PyPrep toolbox (v0.4.3) (Appelhoff et al., 2025). Data segments contaminated by muscle activity or other noise were then manually identified and removed. Independent component analysis (ICA) using the FastICA algorithm (Hyvärinen & Oja, 2000) was then applied to data concatenated over Pre and Post time points, separately for each session (Fig. 1, left). Examination of topographies and time series was used to identify and subsequently remove components associated with blinks, saccades, or ECG. After preprocessing of EEG data, the duration of recordings retained for analysis was comparable between the pre-drug (average: of 281 s; range: 208–342 s) and post-drug (average: 293 s; range: 172–503 s) timepoints (*t*_paired_ = -1.0, *P* = 0.3). These durations are consistent with standard practice in spectral analysis, and exceed durations previously shown to effectively support empirical mode decomposition (EMD) and cycle-based measures (e.g., Fabus et al., 2021).

#### 2.3.2 Spatial filtering & component identification

The conflation of source activity driven by volume conduction is an important consideration for the current study, as this spatial mixing of activity can confound interpretation of the shape of an oscillation examined in sensor space. An approach to address this issue is to apply spatial filters that separate activity derived from different sources. Applying spatial filters corresponds to data-driven re-referencing. An attractive technique for our use case is spatio-spectral decomposition (SSD) (Nikulin et al., 2011), which enhances SNR of specific oscillatory components in the band of interest. As waveform asymmetry is correlated with SNR, applying SSD enables improved detection of waveform features (Schaworonkow & Nikulin, 2019). Within the current study, this was achieved using an approach recently established by Bender et al. (2025). This involved spectral analysis and parameterization, application of SSD to separate potential sources of interest, and source reconstruction and template matching to identify likely anatomical origins for each source. These steps are detailed below.

##### 2.3.2.1 Spectral analysis and parameterisation

Pre-processed, broadband data recorded at centroparietal electrodes (C1, Cz, C2, CP1, CPz, CP2, Pz) were split into 10 s epochs prior to spectral analysis with the multi-taper method (2–40 Hz). These data were then separated into periodic and aperiodic components using the *specparam* toolbox (v1.1.0) (Donoghue et al., 2020) (Fig. 1, left). Spectral activity was modeled with peak width limits of 1 and 12 Hz, a maximum of 6 peaks, minimum peak height of 2 dB, and fixed aperiodic mode. Model fit for all spectra was assessed using *r*^2^ and error values, which revealed consistently high performance (mean *r*^2^ = 98.3 ± 0.03, mean error = 0.03 ± 0.01). Individual alpha peak frequency was defined as the center frequency for the largest peak in the 7–14 Hz range, collapsed across the 6 examined electrodes. Parameterization was performed separately for data from each time point and session.

##### 2.3.2.2 Spatio-spectral decomposition (SSD)

SSD was applied to decompose the data into its underlying sources, from which alpha shape was subsequently derived (Fig. 1, middle). We opted to use SSD for source reconstruction for two reasons. First, it maximizes spectral power within a band of interest, while minimizing spectral power in flanking bands, improving SNR of specifically narrowband oscillations. Second, SSD only requires the time series, avoiding the need to provide a leadfield and anatomical information as inaccuracies here may propagate to subsequent waveform metrics.

For the current study, SSD was applied to data from all available electrodes, concatenated over Pre and Post time points, separately for each session. The band of interest was defined for each participant as the individual alpha peak frequency ± 2 Hz, averaged over Pre and Post time points within each session. The flanking frequencies were then defined as the 2 Hz bands either side of the band of interest (Nikulin et al., 2011). That means, if the individual peak frequency is 10 Hz, 8–12 Hz will be used as the individual band of interest, with 6–8 and 12–14 Hz as the flanking bands (Fig. 1, middle). While the individual alpha peak frequency was estimated using selected centroparietal sensors for computational efficiency, the band of interest is wide and will capture subject-specific alpha-band rhythms even if there are deviations in peak frequency between posterior and centroparietal sources. The filters estimated by SSD were then applied to the broadband-filtered data to obtain time-series for each component. Additionally, spatial patterns for each component were calculated according to Haufe et al. (2014), which provide an estimate of spatial origin of the component. The spatial patterns are used subsequently for template matching using the leadfield from a forward model (see next section). To ensure the presence of an oscillatory peak, and to identify possible slight deviations in the associated peak frequency, each time series generated by SSD was submitted to spectral parameterization (as detailed in the previous section). Only components having an oscillatory peak in the alpha range and SNR exceeding the group-level median (3.87 dB) were considered further to ensure reliable presence of an alpha-rhythm and therefore reliable measurement of alpha waveform shape. In the final dataset, each participant contributed an average of 2.7 ± 1.4 and 2.3 ± 1.4 alpha components to the placebo and diazepam sessions, respectively (unpaired *t*-test, *P* = 0.4).

##### 2.3.2.3 Template matching of SSD components

A forward solution was computed with the MNE *make_forward_solution* function, using a boundary element model and source space derived from the *fsaverage* template data (available as precompiled files within the MNE package), *mindist* = 5 mm, and fixed dipole orientation. The absolute cosine distance between the spatial patterns generated by SSD, and the spatial patterns from the forward model was then calculated (Fig. 1, middle). The component location was defined as the position where this distance was minimized, and only components that fit with a distance < 0.15 were considered. These locations were then categorized according to their cortical lobe using the HCP-MMP1 parcellation (Glasser et al., 2016). As only a single source was localized to the frontal cortex, data from this area were not considered in the analysis.

#### 2.3.3 Waveform shape analysis

The shape of oscillatory activity within the time series extracted from each SSD component was quantified using a previously established pipeline involving two steps. First, application of EMD derived narrow band modes in the alpha range. Although SSD can be expected to generate a more narrowband signal, we further sought to optimize the isolation of individual signal contributions, resulting in more specific waveform metrics. Second, calculation of phase-aligned instantaneous frequency from the alpha intrinsic mode function (IMF) allowed the shape of individual oscillatory cycles to be described (Opie et al., 2024; Quinn, Lopes-dos-Santos, Huang, et al., 2021) (Fig. 1, right).

To reduce the impact of mode mixing (Deering & Kaiser, 2005; Huang et al., 1999), the masked version of EMD was applied using the *mask_sift* function of the EMD toolbox (v0.8.1) (Quinn, Lopes-dos-Santos, Dupret, et al., 2021). A maximum of 6 IMFs were generated using masking frequencies of 120, 64, 32, 11, 7, and 2 Hz. All subsequent analyses focused on the alpha mode. First, fit quality was assessed via visual inspection compared with the raw SSD component time series. Individual alpha cycles were then identified based on the instantaneous amplitude and phase of the signal, derived using the normalized Hilbert transform. To avoid potentially confounding effects of variations in amplitude, only large amplitude cycles were included (top 25^th^ percentile). Cycles were additionally required to have unique control points (i.e., ascending/descending zero crossing, peak, trough; Quinn, Lopes-dos-Santos, Huang, et al., 2021) and not show phase reversals. Using these criteria, a total of 61,310 cycles were identified. The number of cycles contributed by each participant within each condition and time point is detailed in Supplementary Table S1. Cycle numbers were not different between time points for either placebo (*t*_paired_ = -1.3, *P* = 0.19) or diazepam (*t*_paired_ = 1.4, *P* = 0.17) sessions.

Instantaneous frequency (IF) of identified cycles was then quantified as the first derivative of the instantaneous phase with respect to time (Huang et al., 2009). To facilitate comparison of cycles across consistent features (i.e., ensure peaks are compared with peaks), IF values were then projected to a common phase space, producing phase-aligned IF (IF*_PA_*) values (Fig. 1, right). To facilitate comparison across conditions with different mean instantaneous frequency, values were normalized by the mean IF*_PA_* averaged across phase. To characterize variation in alpha shape across individual cycles, IF*_PA_* values were subsequently decomposed using principal component analysis (PCA) (Fig. 1, right). The resulting principal components (PCs) provide a series of waveform motifs that each describe major sources of variance relative to the mean IF profile, with larger scores indicating shapes that are further from the mean. PCA was applied using the *sails* toolbox (v 1.7.0) (Quinn & Hymers, 2020) on demeaned data that was concatenated over subjects and sessions. Consistent with previous work (Quinn, Lopes-dos-Santos, Huang, et al., 2021), split-half reliability testing (500 splits) was applied to validate the response to PCA (see Supplementary Fig. S1). The first four PCs explained >95% of variance in the data and were, therefore, used for subsequent statistical analysis.

### 2.4 Statistical analysis

#### 2.4.1 Spectral data

Parameterized spectral data (aperiodic slope, alpha peak frequency, and power) averaged over centroparietal electrodes were compared between *Sessions* (Placebo, Diazepam) and *Time* (Pre, Post) using Bayesian generalised linear mixed models (GLMM). For alpha power and peak frequency data in the diazepam session, one participant was missing data in the Post time point, whereas another participant was missing data at Pre and Post time points. While GLMM’s can model missing data, they must be missing at random. As it was unclear if missing alpha values could be considered random, these spectral data were imputed prior to statistical analysis with multiple imputation (20 imputed datasets) in R using the *mice* package (Van Buuren & Groothuis-Oudshoorn, 2011). Missing data were imputed using the multiple imputation by chained equations approach, the details of which are described by Azur et al. (2011). This involves repeated cycles of regression to identify reasonable estimates of imputed values. This process is then repeated a predefined number of times (20 in the current study) to generate multiple imputed datasets. The model of interest is applied to each of the imputed datasets, and outcomes are inferred by collapsing over models. Data were modeled with a skew-normal distribution and identity link function, with maximal by-participant random effects for time (model 1). Each model was run using 8 independent chains, with 1000 warm-up and 3000 post-warm-up samples (totaling 480,000 post warm-up samples across the 20 imputed datasets), and default flat priors.

Spectral Data ~ Session*Time+(1+Time |Subject) (model 1)

#### 2.4.2 Shape data

Bayesian GLMMs were also used to assess changes in alpha shape over *Time* (Pre, Post) and between *Lobes* (Occipital, Temporal, Sensorimotor, Parietal) and *Sessions* (Placebo, Diazepam). A single model was run for each PC of interest: each model incorporated all data that met the quality control measures defined above, and used a student’s *t* distribution with identity link function. However, between-session variance in the presence of some oscillatory activity meant it was not possible to match SSD components between sessions. Consequently, the specific participant cohort included within each session was not matched, and we were unable to make direct comparisons between data from the placebo and diazepam sessions. In an attempt to address this limitation, the model implemented maximal by-participant random effects for both time and SSD component (model 2). Nonetheless, within-session contrasts were sufficient to address the study’s main aims. Each model was run using 4 independent chains, with 1000 warm-up and 3000 post-warm-up samples (totaling 12,000 post-warm-up samples), and weakly informative priors (Normal (0, 1)) for fixed effects.

PC ~ Session*Time*Lobe+(1+Time+SSD | Subject) (model 2)

To further investigate drug-related shifts in the distribution of PC scores, additional comparisons across Pre and Post time points were made using two-sample Cramér-von Mises tests, with bootstrapped 95% confidence intervals (10,000 resamples) also generated to facilitate population-level inference. Both tests were implemented in the *SciPy* toolbox (v1.14.1) (Virtanen et al., 2020). A Bonferroni correction was applied to adjust for multiple comparisons in these data, with *P* < 0.003125 (i.e., 0.05 / [4 principal components * 4 cortical lobes]) considered significant.

#### 2.4.3 Bayesian estimation

All GLMMs were computed in R (v 4.4.3) using RStudio (v2024.12.1). Posterior distributions were estimated within BRMS (Bürkner, 2017), using the No-U-Turn Sampler (NUTS) extension of Hamiltonian MCMC. Chain convergence was assessed using Rhat values < 1.1 and posterior predictive checks (Gabry et al., 2019; Gelman & Rubin, 1992). Custom contrasts of main effects and interactions were generated using the *emmeans* package (Lenth, 2025), with effect existence and significance identified using the probability-of-direction (e.g., *pd*; Makowski et al., 2019) and region of practical equivalence (ROPE), respectively. For the current study, ROPE was defined as ± 5% of the standard deviation for the data being compared (Kruschke, 2018). The null hypothesis of no difference was accepted for comparisons where the 89% highest density interval (HDI) fell completely inside the ROPE (i.e., 100% in ROPE). In contrast, the null hypothesis was rejected when the 89% HDI fell completely outside the ROPE (i.e., 0% in ROPE). No decision was made if the 89% HDI partially overlapped ROPE (Kruschke, 2018; Moore et al., 2025; Opie et al., 2024). Results for all Bayesian models are presented as median values [lower 89% HDI, upper 89% HDI].

## 3 Results

### 3.1 Drug-related changes in spectral data

**Fig. 2.**
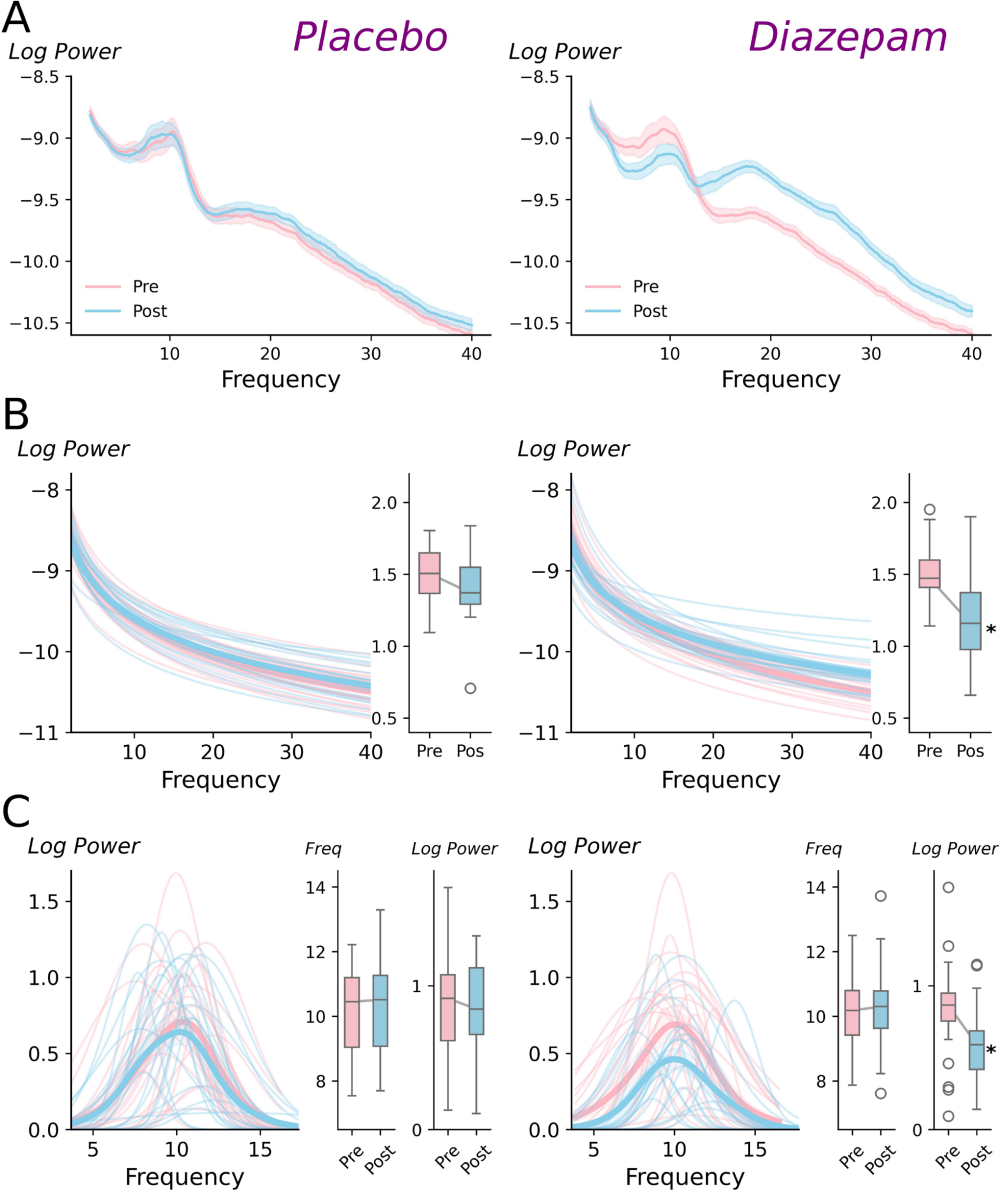
Effects of diazepam and placebo on periodic and aperiodic spectral features (A) Power spectral density curves (log(V^2^/Hz) recorded before (Pre) and after (Post) intake of placebo (left) or diazepam (right). Shaded area shows ± 1 standard error of the mean (B) Aperiodic components (line plot) and slopes (box plot) plotted before (Pre/ pink) and after (Post/ blue) intake of placebo (left) or diazepam (right). (C) Periodic components (line plot), alpha peak frequency (middle box plot), and alpha power (right box plot) recorded before and after placebo (left) or diazepam (right). For panels B & C, thick lines show group average and thin lines show responses for individual participants. Furthermore, lower, middle, and upper lines of boxplots indicate 25^th^, 50^th^, and 75^th^ percentiles, respectively, whiskers extend to 1.5 x the interquartile range. *pd > 99%, 0% in ROPE, relative to *Pre*.

First, to align our results to previous literature, we compared spectral characteristics between sessions and time points (Fig. 2). At baseline, between-group comparisons for all measures were inconsistent and failed to provide sufficient evidence to accept or reject the null hypothesis (all *pd* < 80%, % in ROPE: 29.7–41.7%). For all measures in the placebo session, in addition to alpha frequency in the diazepam session, within-group comparisons were also inconsistent and failed to provide sufficient evidence to accept or reject the null hypothesis (all *pd* < 86%, % in ROPE: 21.0–44.9%). In contrast, diazepam was associated with consistent and significant reductions in both aperiodic slope (*pd* = 99%, 0% in ROPE; Fig. 2B, right) and alpha power (*pd* = 99%, 0% in ROPE; Fig. 2C, right) at the Post time point.

### 3.2 Drug-related changes in alpha waveform shape

**Fig. 3.**
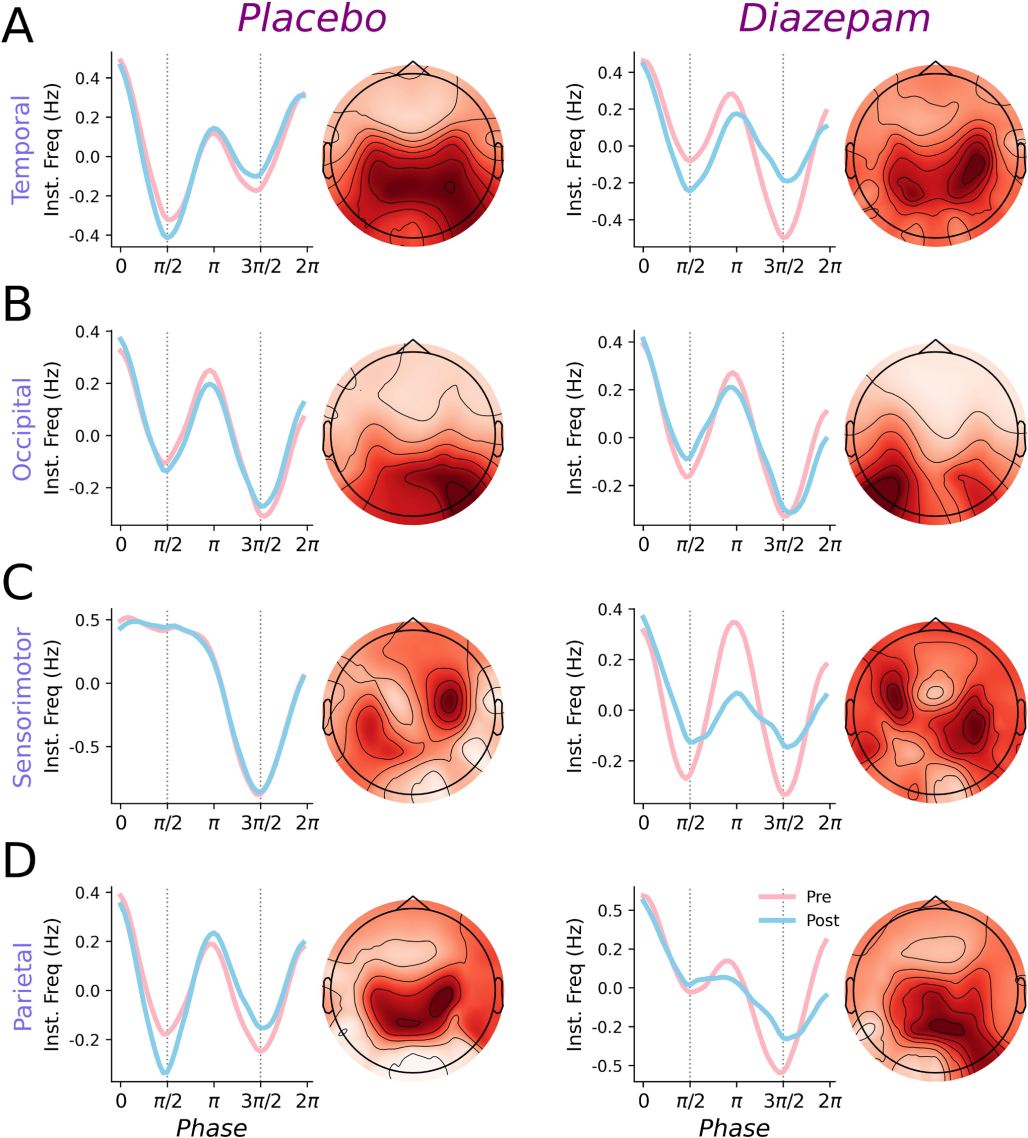
Effects of diazepam and placebo on IF_PA_ profiles in each cortical area. Group-averaged IF_PA_ profiles showing the deviation from mean instantaneous frequency (line plots; pre in pink, post in blue) and spatial patterns associated with the included SSD components (topoplots) for temporal (A), occipital (B), sensorimotor (C), and parietal (D) cortices. Responses from the placebo session are shown in the left column, whereas responses from the diazepam session are shown in the right column. Vertical dashed grey line in IF_PA_ profiles indicates timing of cycle peak (left) and trough (right).

**Fig. 4.**
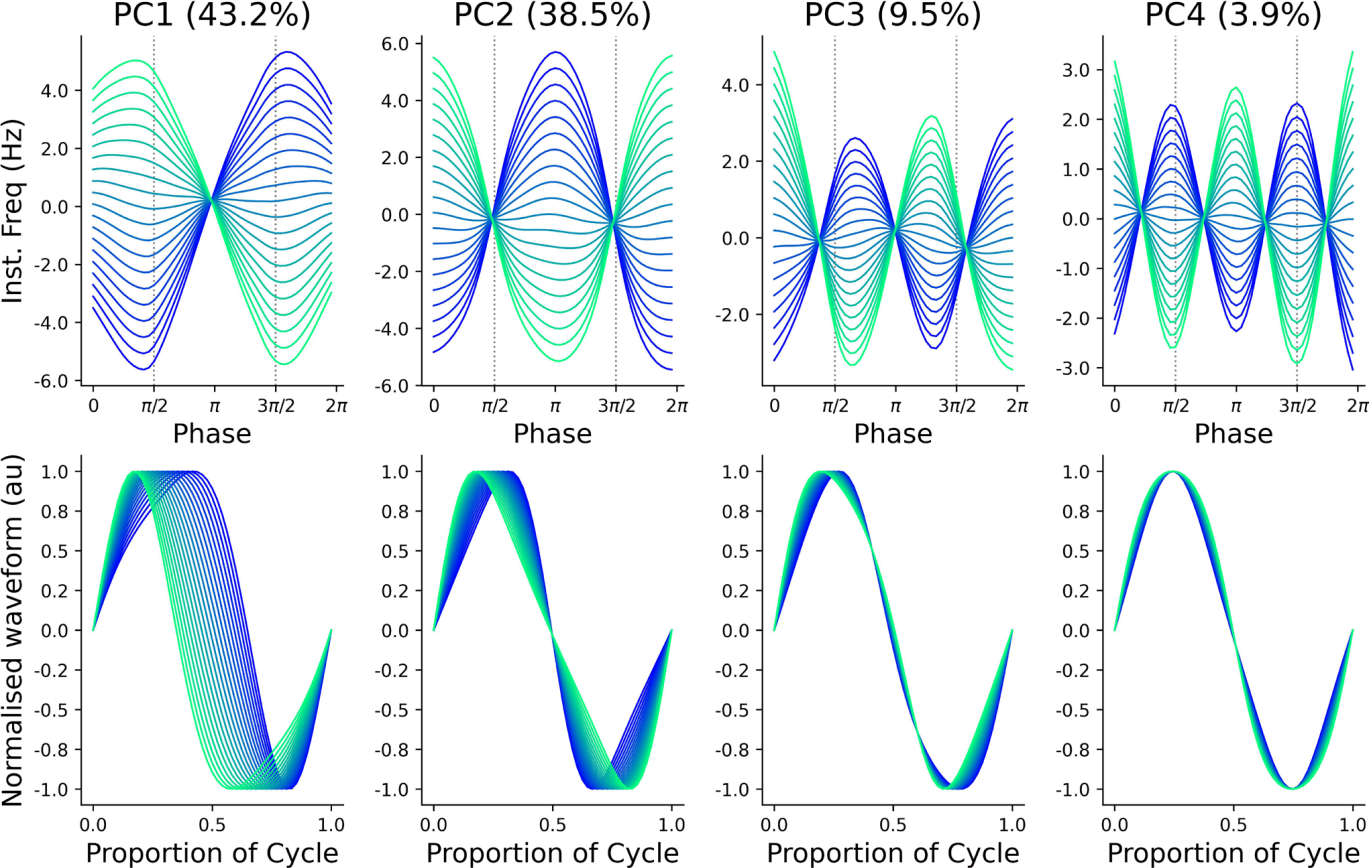
Waveform motifs identified with PCA. IF_PA_ profiles showing the deviation from mean instantaneous frequency (top row) and associated normalized waveforms (bottom row) for the top four principal components (PC, percentage indicates proportion of variance explained), plotted across the percentiles of scores observed for all alpha cycles. Color gradient ranges from 1^st^ (blue) to 99^th^ (green) percentile. Vertical dashed grey line in top row indicates timing of cycle peak (left) and trough (right).

Having established directionality of changes in spectral measures, we now turn to waveform shape analyses: for each cortical area of interest, Figure 3 shows IF*_PA_* profiles recorded before and after placebo and diazepam, in addition to the spatial patterns of the underlying alpha components. Across areas, IF*_PA_* profiles tended to indicate that alpha peaks and troughs were associated with reduced frequency, whereas zero crossings were associated with increased frequency. To quantify these changes, PCA was used to decompose IF*_PA_* profiles into waveform motifs; the first 4 components derived from this process explained more than 95% of variance in the data and are characterized based on their IF*_PA_* and normalized waveforms in Figure 4. These components explained more than 95% of variance in the data, although the majority was partitioned within PC1 and PC2. PC1 (~43% of variance) reflected peak-trough width asymmetry (negative scores: broad peaks/narrow troughs, positive: narrow peaks/broad troughs). PC2 (~38% of variance) reflected edge-speed asymmetry (negative: slow ascending/fast descending edges, positive: fast ascending/slow descending edges). Subsequent PCs explained less variance and involved more subtle cycle features: PC3 (~9 % variance) described asymmetries involving speed of the rising edge (negative: slower, positive: faster) and curvature of the descending edge (negative: convex, positive: concave), whereas PC4 (~4%) described symmetrical changes in extrema width (negative: narrower, positive: broader).

**Fig. 5.**
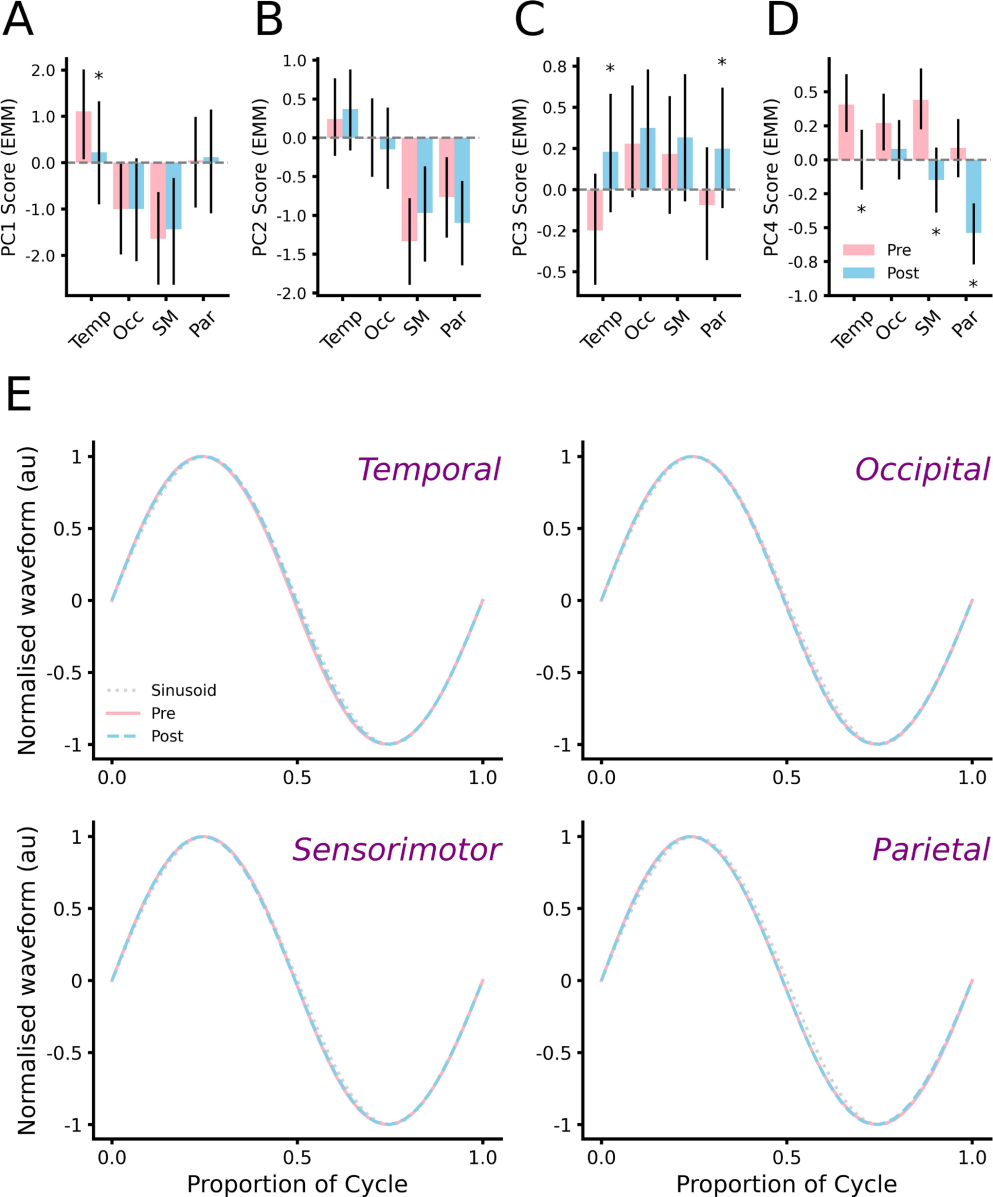
PC scores identify subtle changes in alpha shape. (A–D) Estimated marginal means from Bayesian GLMMs showing mean scores for PC1 (A), PC2 (B), PC3 (C), and PC4 (D) before (pink) and after (blue) diazepam. Temp: temporal, Occ: occipital, SM: sensorimotor, Par: parietal. Error bars show 89% HDI. (E) Normalised waveforms associated with median PC scores for each cortical area recorded before (pink curve) and after (blue curve) diazepam. Sinusoid plotted for reference (grey dotted curve). *pd > 99%, 0% in ROPE, relative to *Pre.*

To quantify drug-related changes in alpha shape, scores for the first 4 PCs were compared between time-points, with results plotted separately for each cortical area in Figure 5A–D and Supplementary Figure S2. Diazepam was associated with consistent and significant reductions in scores for PC1 in the temporal cortex (*pd* = 99%, 0% in ROPE; Fig. 5A), and PC4 in temporal (*pd* = 99%, 0% in ROPE), sensorimotor (*pd* = 100%, 0% in ROPE), and parietal (*pd* = 100%, 0% in ROPE) cortices (Fig. 5D). Furthermore, consistent and significant increases in PC3 scores were found in both temporal (*pd* = 100%, 0% in ROPE) and parietal cortices (*pd* < 99%, 0% in ROPE; Fig. 5C). While comparisons of PC4 in occipital cortex also showed consistent changes after diazepam (pd = 97.8%), this failed to reach a practical level of significance (8.8% in ROPE). All other comparisons were inconsistent and failed to provide sufficient evidence to accept or reject the null hypothesis (all *pd* < 96.6%, % in ROPE: 16.6–87.8%; Supplementary Fig. S2). To illustrate how diazepam-related changes in PC scores influenced alpha shape, normalized waveforms associated with the median score for each component are plotted in Figure 5E.

**Fig. 6.**
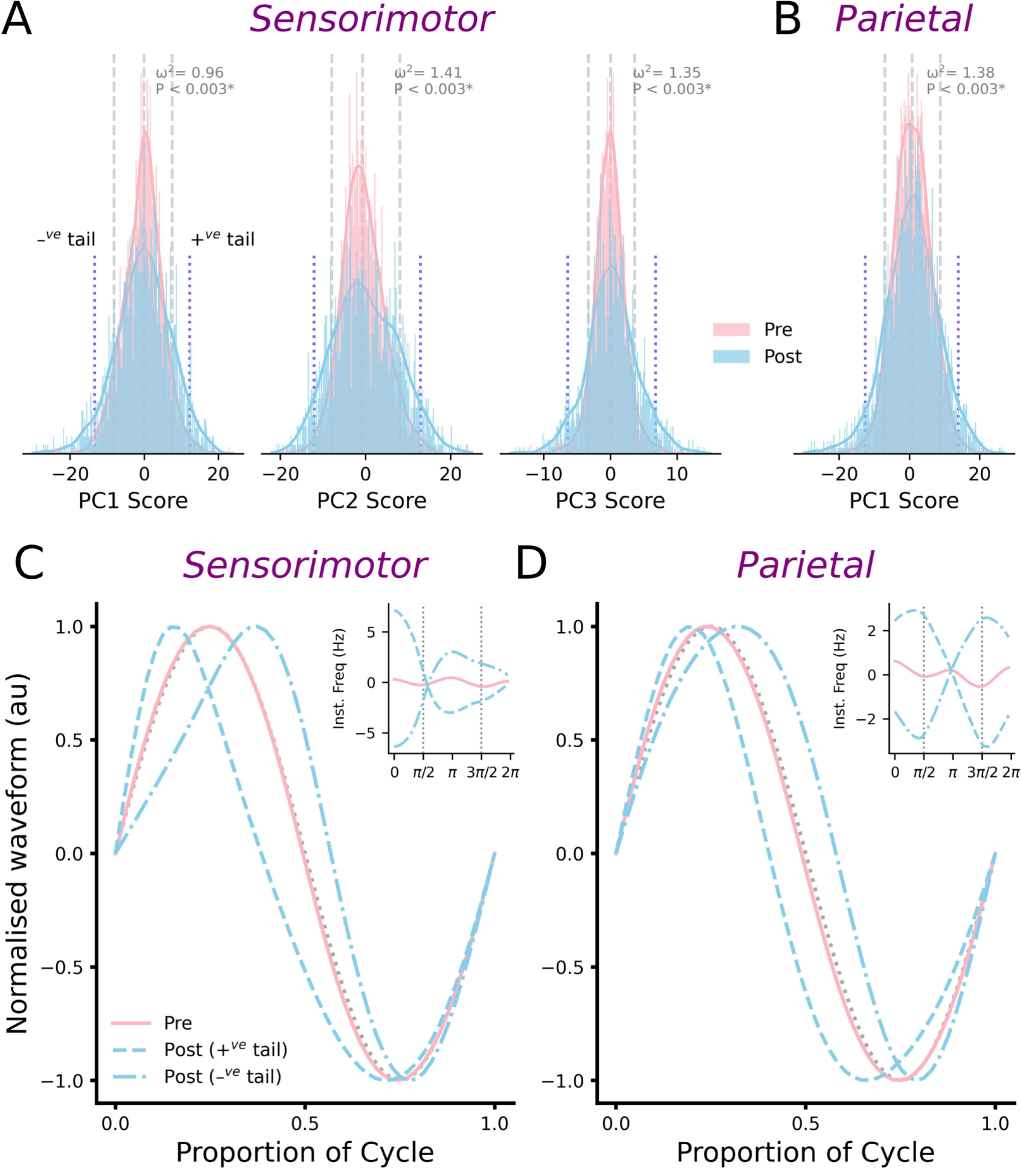
Extreme alpha shapes are increased after diazepam. (A-B) Probability densities for PC scores from sensorimotor (A) and parietal (B) cortices, which showed significant changes in distribution due to diazepam. Vertical dashed grey lines indicate 10^th^, 50^th^, and 90^th^ data percentiles. Post-diazepam alpha tended to have an increase in scores in the top and bottom 10^th^ percentiles. (C-D) Normalized waveforms associated with diazepam-related changes in score distribution. Waveforms were derived using scores identified by vertical dotted blue lines in panels A and B (denoted +^ve^ tail and –^ve^ tail in panel A). For PCs failing to show significant change in distribution, scores were set at the median value. Inset shows IF_PA_ profiles for each curve, with a vertical dashed grey line indicating timing of cycle peak (left) and trough (right).

We examined the changes in score distribution across Pre and Post conditions via two-sample distributional testing for each PC. To facilitate data interpretation, distributions showing significant drug effects are plotted in Figure 6, whereas all other distributions are plotted in Supplementary Figures S3 and S4. For the diazepam session, there were significant differences in score distribution at the post time point for PC1 (ω^2^ = 0.96, *P* = 0.003, 95%CI*_Boot_* = [0.37, 1.91]), PC2 (ω^2^ = 1.41, *P* = 0.0003, 95%CI*_Boot_* = [0.67, 2.43]) and PC3 (ω^2^ = 1.35, *P* = 0.0004, 95%CI*_Boot_* = [0.45, 2.95]) in the sensorimotor cortex (Fig. 6A), in addition to PC1 in the parietal cortex (ω^2^ = 1.38, *P* = 0.0004, 95%CI*_Boot_* = [0.51, 2.75]) (Fig. 6B). For these components, changes in distribution consistently involved an increase in more extreme scores (i.e., bottom and top 10^th^ percentile). Normalized waveforms associated with these more extreme scores are shown in Figure 6C and 6D. All other comparisons failed to show any significant change in distribution (Supplementary Figs. S3 & S4).

## 4 Discussion

While examination of neural oscillations generally focuses on spectral features such as power, frequency, and phase, a developing literature has identified oscillation shape as providing additional neurophysiological insight. However, the link between oscillation shape and specific neural mechanisms such as neurotransmission has not been established in non-invasive human recordings. Within the current study, we began to address this underexplored field by investigating changes in the shape of alpha oscillations induced by potentiation of GABA_A_-mediated inhibitory neurotransmission. This was achieved by quantifying alpha shape derived from resting-state EEG recordings before and after intake of diazepam or placebo. Using this approach, an initial analysis found significant changes in PCs that described subtle shifts in alpha shape, the specific nature of which varied between cortical areas. However, additional analysis focusing on alterations in the distribution of PC scores identified stronger effects of diazepam that were limited to alpha cycles originating from sensorimotor and parietal cortices. These results provide the first evidence that, when assessed non-invasively with EEG, GABA_A_ergic processes can be indexed via the shape of alpha oscillations.

### 4.1 Spectral effects of diazepam

Prior to waveform analysis, effects of increasing neurotransmission through GABA_A_ receptors were examined using a conventional spectral decomposition technique that involved Fourier methods (i.e., the multi-taper method). Consistent with findings from previous studies (for review, see; Lozano-Soldevilla, 2018), this analysis identified a significant reduction in alpha power without any change in frequency (Fig. 2). These effects have been suggested to reflect drug-related changes in neuronal firing that do not influence oscillatory frequency (e.g., reduced spikes per burst without a change in burst frequency), or alterations to thalamic input to cortical alpha generators (Lozano-Soldevilla et al., 2014). Irrespective of the specific mechanism, reductions in alpha power demonstrate that diazepam was effective at producing the expected changes in oscillatory activity. However, we additionally observed a drug-related reduction in the aperiodic component, corresponding to a flattening of the PSD (Fig. 2). Previous simulation work has suggested that the aperiodic slope is an index of the balance between neural excitation and inhibition (Gao et al., 2017); while the drug-related changes observed here are consistent with this idea, the direction of change was not. Specifically, current interpretations of aperiodic slope suggest potentiation of inhibitory function should drive an increase in slope, as opposed to the reduction we found. However, it is important to note that the nature of the relationship between aperiodic activity and neural activity is still being clarified (Donoghue, 2024). In particular, recent studies using pharmacological intervention or ethanol consumption to potentiate GABAergic function have reported both increased (Barone et al., 2025; Gonzalez-Burgos et al., 2023; Salvatore et al., 2024; Stock et al., 2020; Waschke et al., 2021) and decreased (Barone et al., 2025) aperiodic activity. This includes contradictory responses to the same drug (i.e., diazepam; current study vs*.* Gonzalez-Burgos and colleagues) and drug class (i.e., positive allosteric modulators of GABA_A_ receptors; current study vs*.* Barone and colleagues). While methodological differences possibly contributed to this variability, these outcomes, nonetheless, demonstrate that further work is needed to clarify the neurophysiological interpretation of aperiodic activity.

### 4.2 Diazepam alters the shape of alpha oscillations

To examine drug-related changes in alpha shape, the current study applied a recently developed waveform analysis pipeline (Quinn, Lopes-dos-Santos, Huang, et al., 2021). This involved derivation of IF*_PA_* profiles for individual cycles, and subsequent decomposition with PCA to extract motifs describing key features (e.g., Figs. 3 & 4). Consequently, changes in PC scores over time can be interpreted as changes to specific facets of alpha shape. Using this approach, we found that scores for the top four PCs were not altered in the placebo session, and this was consistent across all cortical areas. In contrast, diazepam was associated with significant changes in PC1, PC3, and PC4, the nature of which differed between cortical areas: while changes in PC1 were limited to temporal cortex, effects on PC3 were apparent in both temporal and parietal cortices, whereas PC4 was altered in all cortices other than occipital (Fig. 5A–D). It is also interesting to note that larger effects were apparent for PCs explaining less variance, which could suggest sensitivity to noise. Despite this, no comparable changes were apparent in the placebo condition, which would have been expected if driven by noise. Taken together, our results therefore demonstrate that examination of oscillation shape can reveal regionally specific effects of GABA_A_-mediated inhibitory neurotransmission on alpha cycles. However, while these changes were significant, it is important to note that their impact on shape was highly subtle. This is demonstrated in the normalized waveforms plotted for each cortical area in Figure 5E. Within these, all waveforms show subtle deviations in shape with respect to a sinusoid which are consistent with the known non-sinusoidal shape of alpha cycles (Cole & Voytek, 2017). While differences between Pre and Post waveforms are only modest, it is important to note the macroscale nature of the EEG recordings from which these data were derived. Consequently, these modest differences do not necessarily preclude changes in neurophysiological dynamics.

Despite this, the ‘shape space’ described by each PC is large and variable. Furthermore, recent work demonstrates that functionally significant changes in shape may be limited to sub-populations of cycles. Specifically, performance of reaching movements was associated with changes in the shape of beta oscillations that were limited to cycles having more extreme shape (i.e., scores furthest from the median) (Rayson et al., 2023; Szul et al., 2023). Consequently, the possibility remained that different effects of diazepam may be apparent within specific sections of the score distribution. In support of this, subsequent inspection of score distributions identified several significant changes following diazepam that were not apparent with placebo. In contrast to our primary analysis, these were specific to cycles originating from sensorimotor and parietal cortices, involved a different collection of PCs, and an increase in the proportion and heterogeneity of cycles within the tails of the distribution (i.e., more extreme shape; Fig. 6A, B). Taken together, these results suggest that, although waveform shape can index important neuromechanistic processes, the way in which this is quantified needs further consideration. In particular, much greater exploration of different segments of the ‘shape space’ is required. An important consideration in achieving this will be to consider alternative decomposition approaches that might allow more granular description of the hugely variable ‘shape space’. Elegant examples of how this might be achieved were recently reported by work using non-linear dimensionality reduction (Lee et al., 2021; Sebastian et al., 2023) in conjunction with the clustering techniques (Lee et al., 2021). Investigating if similar approaches have utility in non-invasive human recordings will be an exciting area for future work. In addition, the discrepancy between GLMM and distributional tests demonstrates that the way in which PC data are statistically examined needs optimization. Given that GLMM assumes that the data distribution does not change over time, whereas the developing literature (Rayson et al., 2023; Szul et al., 2023) indicates that changes in distribution are a consistent response of these data, distributional tests may be preferred in future studies.

### 4.3 Spatially specific effects of diazepam on alpha shape

Although sensorimotor and parietal cortices both demonstrated an increase in alpha cycles with more extreme shape and heterogeneity following diazepam, the specific changes in shape differed between areas: while effects on sensorimotor cycles included changes in peak width and edge speed, effects on parietal cycles were more specific to alterations in peak-trough asymmetries (Fig. 6C, D). One reason for this differential response to diazepam could be that it reflects unique features of the neural circuits generating alpha oscillations in each area. Indeed, recent work using both animal and human recordings has suggested this as an explanation for regional variations in oscillation shape (García-Rosales et al., 2024; Giehl & Siegel, 2024). However, the mechanisms driving alpha generation are complex and not well understood. For example, the extent to which alpha is supported by sources in different locations (local cortical mechanisms vs. thalamic mechanisms), or involves different cell types (primarily interneuron generation [c.f. gamma-activity] vs. pyramidal neuron-interneuron interplay) remains an area of active investigation (for review, see: Lozano-Soldevilla, 2018). It is, therefore, important to note that a multitude of factors may have given rise to the spatially distinct waveform metrics observed here.

Nonetheless, the pharmacological manipulation we investigated does support some mechanistic speculation into alpha generation in different cortical regions, specifically with respect to variations in GABA_A_ receptors. Although they are located throughout the brain, GABA_A_ receptors are highly heterogeneous in nature. In particular, there are four GABA_A_ receptors that are sensitive to diazepam; these are defined by different α subunits (α1-3 & 5), the presence of which determines unique anatomical and subcellular distribution, in addition to electrophysiological and functional effects (Engin et al., 2018; Olsen & Sieghart, 2009; Sieghart & Sperk, 2002). For example, while receptors having an α1-3 subunit tend to be located synaptically, those with an α5 subunit are more focused on extrasynaptic membranes (Engin et al., 2018). Consequently, diazepam can be expected to have some influence on both phasic (i.e., synaptically mediated) and tonic (i.e., extrasynaptically mediated) inhibition (Engin et al., 2018), and spatial variance in this effect could contribute to the regional modulation and heterogeneity of waveform shape observed in the current study. Future pharmacological investigation targeting alternative GABA_A_ receptor subtypes—for example by testing zolpidem, a positive modulator mainly at the α1-GABA_A_ receptor—could be an interesting way to further investigate this concept. Modulation of other key neurotransmitter and neuromodulatory systems will also be important. In addition to characteristics of the local generative circuitry, sensorimotor and parietal areas also have unique connectivity networks (e.g., Jann et al., 2010), the idiosyncrasies of which may contribute to regional effects of diazepam on alpha shape. In support of this, recent work reported differential changes in resting-state functional magnetic resonance imaging (fMRI) connectivity in sensorimotor and parietal cortices following administration of alprazolam (Wein et al., 2024), an alternative positive allosteric modulator of GABA_A_ receptors.

It is worth noting that some baseline variance in waveform shape was apparent, with sensorimotor cycles being particularly affected (Fig. 3). As groups were not matched, some variance is to be expected, likely driven by differential contributions from a range of alpha generators. This would have been exacerbated by the large cortical area over which sources were grouped. From the perspective of sensorimotor cycles, this rhythm characteristically includes strongly lateralized sources over the bilateral sensorimotor cortex. Differences in the extent to which these were present may have contributed to the observed variance. This is supported by the spatial patterns shown in Figure 3, which appear to indicate a more bilateral topography for sensorimotor alpha in the diazepam session. As comparisons were not made between sessions, this variance would not have influenced our results. Nonetheless, it further highlights the need for optimization of how waveform shape is examined, particularly in the context of repeated-measures designs.

### 4.4 Limitations

The current study includes some limitations that warrant discussion. First, while a spatial filtering technique was applied to localize different alpha sources, it is important to note that this did not use individualized structural MRI data. As we refrained from fine-grained spatial interpretation of the data, the potential impact of source localization error on the reported outcomes should not be substantial. However, spatial information should, nonetheless, be interpreted with caution. Second, while attempts were made to match alpha sources between sessions within each subject, the nature of the spatial filtering we applied meant this was not possible for the majority of participants. While there was substantial overlap in the participants contributing data to each session (n = 12), our analysis and interpretation of the data was limited to within-subject comparisons. In an attempt to address this limitation to some extent, the GLMMs we implemented included maximal by-participant random effects (i.e., intercepts and slopes) for time and SSD component. However, future work using alternative approaches that allow sources to be matched more specifically will be important for confirming the findings we report here. Third, alpha peak frequency was assessed in a centroparietal subset of electrodes, which could be suggested to bias SSD estimates towards central alpha generators. However, given their generally increased amplitude/SNR, posterior sources will still contribute strongly to centroparietal recordings, and we would, nonetheless, expect to see their peak frequency in the montage we applied (if different from central sources). Furthermore, cycle detection was applied within component time series, after the spatial separation of different source contributions, therefore not pooling across different types of alpha sources. We, therefore, do not expect that generators were reconstructed with disproportionate SNR relative to more occipital components. Fourth, effects of diazepam on behavior (e.g., arousal, attention, motor function) were not quantified, but were likely present. As behavior has been shown to also drive changes in waveform shape (Opie et al., 2024; Quinn, Lopes-dos-Santos, Huang, et al., 2021), it is possible that factors other than direct effects on GABA receptors contributed to the response to diazepam. This will be important to investigate in future studies that also include behavioural indices.

In conclusion, the current study used a pharmaco-EEG approach to investigate the extent to which the shape of alpha oscillations, recorded non-invasively in humans using EEG, can index inhibitory neurotransmission involving GABA_A_ receptors. Subsequently, while all facets of alpha shape were unchanged in the placebo session, demonstrating methodological consistency, ingestion of diazepam—a positive allosteric modulator of GABA_A_ receptors—was associated with significant changes in alpha shape. Importantly, the specific effects of diazepam on alpha shape varied between cortical areas, and included a combination of highly subtle and more clearly discernible effects. These findings provide further support for the idea that oscillation shape is neurophysiologically informative. However, they additionally highlight the need for greater investigation of the ‘shape space’, particularly with respect to identifying the functional and physiological relevance contained within different clusters of the waveform shape space.

## Supplementary Material

Supplementary Material

## Data Availability

Due to the nature of ethical consent obtained, data cannot be shared. All codes to replicate analyses and generate figures from this article can be found at: https://github.com/g-opie/Diazepam-and-alpha-shape
