# Supplementary material for: Diazepam alters the shape of alpha oscillations recorded from human cortex using EEG
Source: Imaging Neurosci (Camb). 2026 Mar 12;4:IMAG.a.1169. doi: 10.1162/IMAG.a.1169 (PMC12983582; doi:10.1162/IMAG.a.1169)
Supplement: Supplementary Material [file IMAG.a.1169_supp.pdf]

Supplementary Table

Table S1. Number of cycles per participant

Participant	Placebo		Diazepam		Total
	Pre	Post	Pre	Post	
1	1091	1320	–	–	2411
2	1007	1029	1859	1460	5355
3	1053	954	1305	1055	4367
4	1196	913	345	388	2842
5	1592	1845	405	674	4516
6	1787	1614	1326	1665	6392
7	–	–	899	540	1439
8	431	508	–	–	939
9	1513	2414	489	275	4691
10	683	525	579	356	2143
11	–	–	353	491	844
12	–	–	–	–	–
13	–	–	–	–	–
14	–	–	–	–	–
15	719	855	1439	1157	4170
16	880	1183	–	–	2063
17	1182	1373	1135	1091	4781
18	2934	3036	838	946	7754
19	869	903	772	516	3060
20	–	–	908	744	1652
21	441	389	517	544	1891
Mean	1158	1257	877	793	

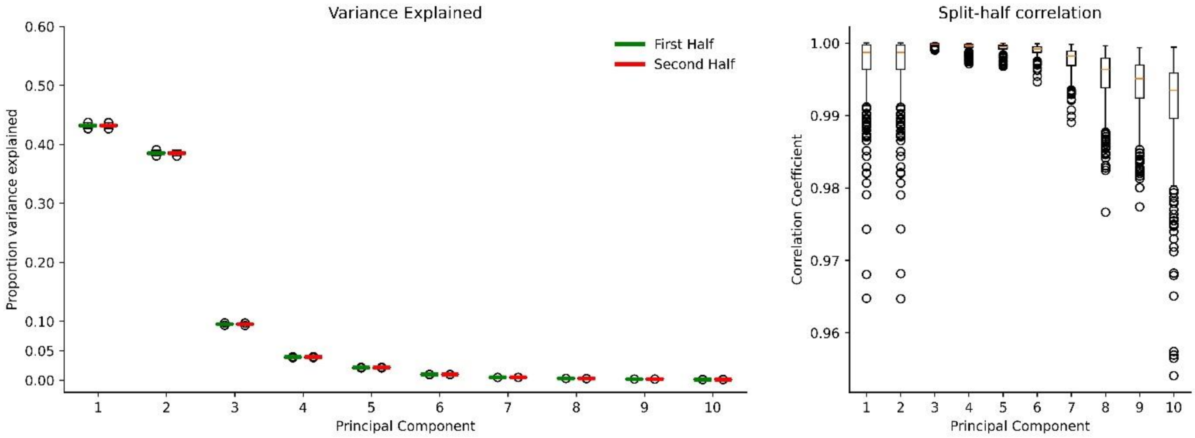

Figure S1. Split-half reliability validation of PCA, showing high consistency in proportion of variance explained for each component and ordering of components. ***(left)*** Variance explained for each principal component, compared between first *(green)* and second *(red)* halves of split. ***(right)*** correlation coefficient between halves for each principal component**.**

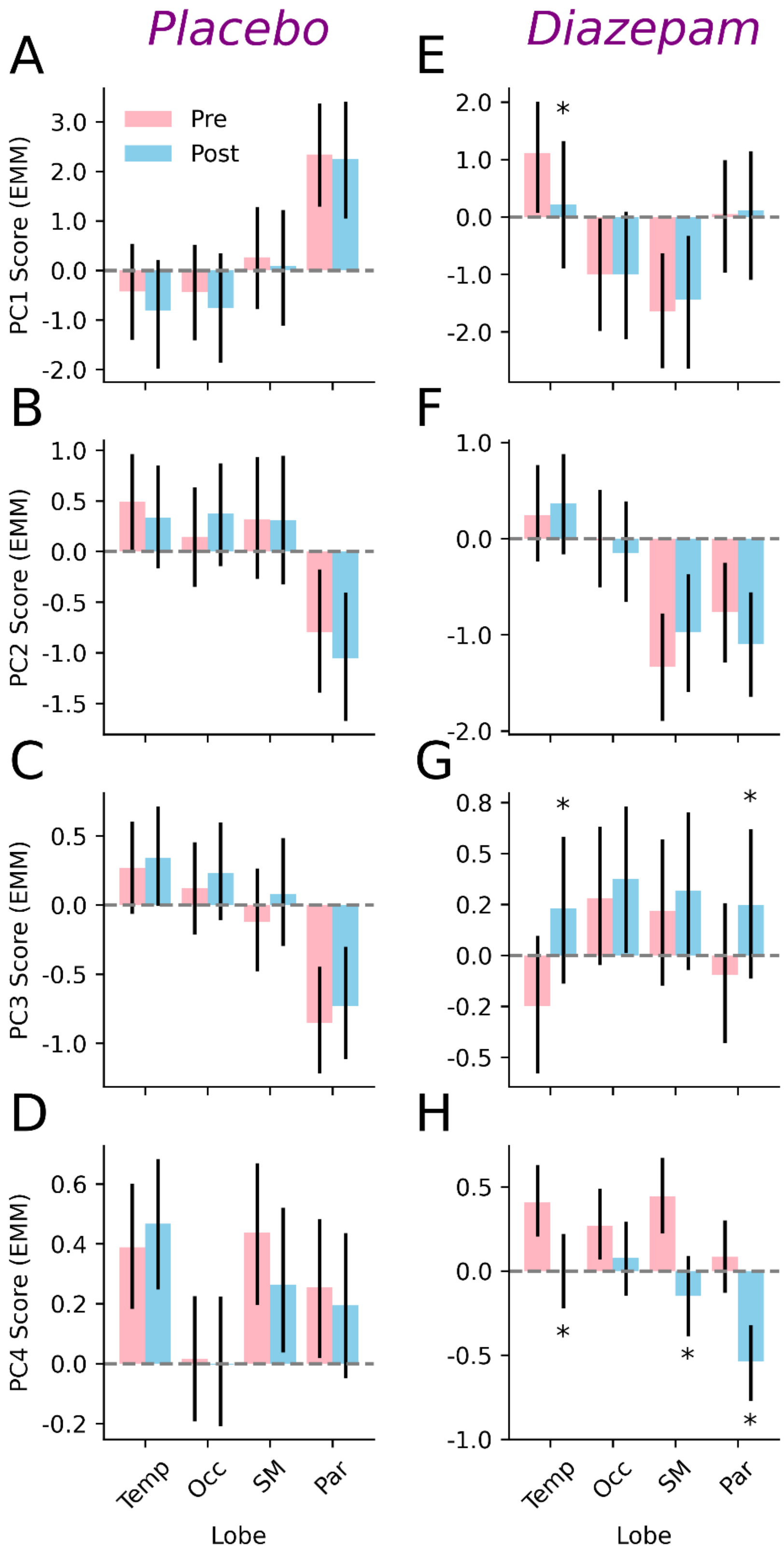

Fig S2. Effects of diazepam and placebo on PC scores. Estimated marginal means from Bayesian GLMMs for top four PCs *(rows)* before *(pink bars)* and after *(blue bars)* placebo ***(A-D)*** or diazepam ***(E-H)***. *pd > 99%, 0% in Rope, compared to *Pre*.

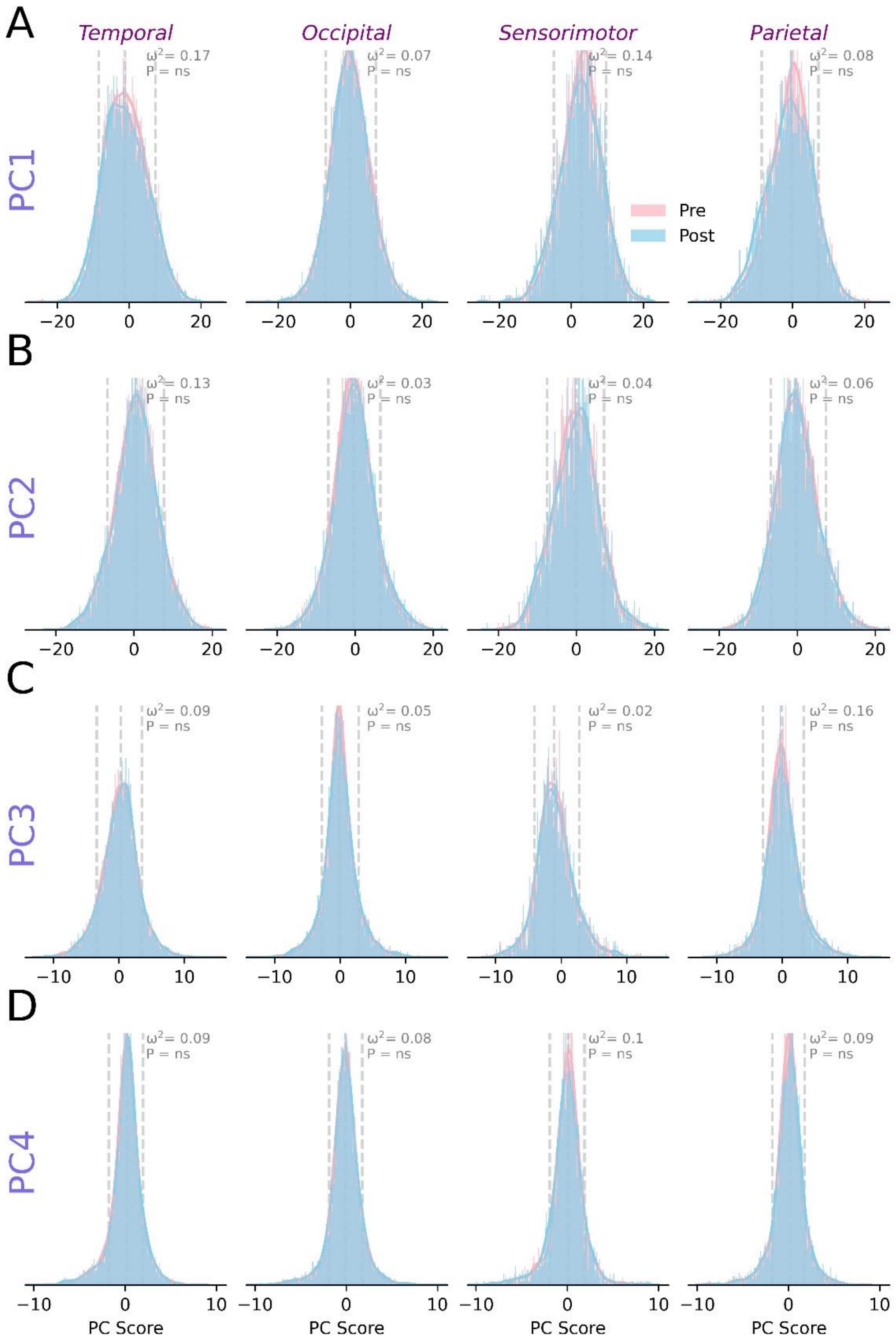

Fig S3. Distribution of PC scores for placebo session. ***(A-D)*** Probability densities for top four PCs before *(pink)* and after *(blue)* intake of placebo, separated by each cortical area *(columns)*. *ns* = not significant, relative to *Pre*.

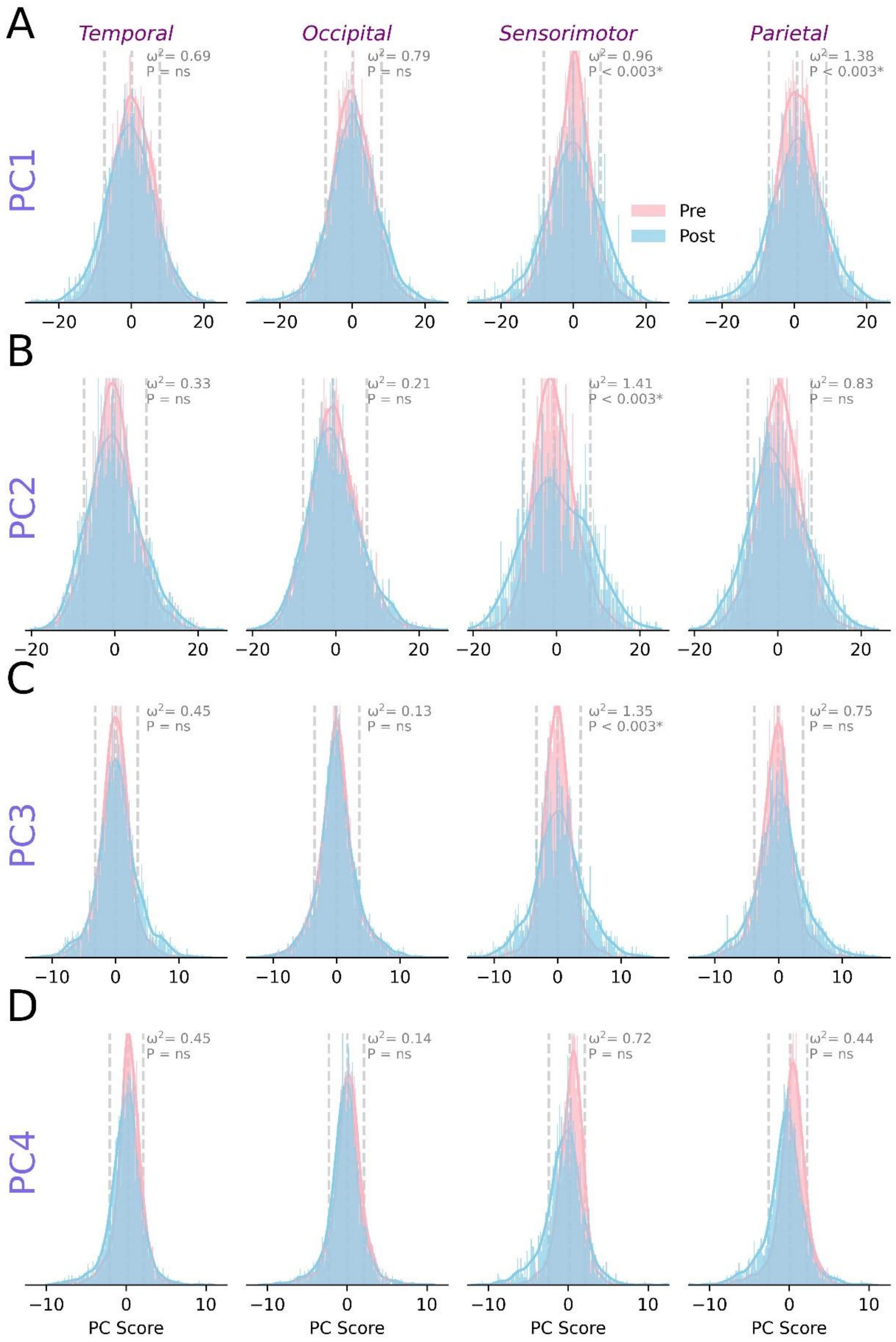

Fig S4. Distribution of PC scores for diazepam session. ***(A-D)*** Probability densities for top four PCs before *(pink)* and after *(blue)* intake of diazepam, separated by each cortical area *(columns)*. *ns* = not significant, relative to *Pre*.
